# Prevalence and Burden of Diabetes-Related Foot Disease in New South Wales, Australia: Evidence from the 45 and Up Study Survey Data Linked with Health Services Data

**DOI:** 10.3390/ijerph182111528

**Published:** 2021-11-02

**Authors:** Moin Uddin Ahmed, Wadad Kathy Tannous, Kingsley Emwinyore Agho, Frances Henshaw, Deborah Turner, David Simmons

**Affiliations:** 1 Translational Health Research Institute, School of Medicine, Western Sydney University, Campbelltown, NSW 2560, Australia; k.tannous@westernsydney.edu.au (W.K.T.); K.Agho@westernsydney.edu.au (K.E.A.); 2 School of Business, Western Sydney University, Parramatta, NSW 2150, Australia; 3 School of Health Sciences, Western Sydney University, Campbelltown, NSW 2560, Australia; frances.henshaw@convatec.com; 4 African Vision Research Institute (AVRI), University of KwaZulu-Natal, Durban 4041, South Africa; 5 ConvaTec, 530 Springvale Rd., Glen Waverley, VIC 3150, Australia; 6 School of Clinical Sciences, Podiatric Medicine, Queensland University of Technology, Brisbane, QLD 4059, Australia; deborah.turner@qut.edu.au; 7 Macarthur Clinical School, Western Sydney University, Sydney, NSW 2560, Australia; Da.Simmons@westernsydney.edu.au

**Keywords:** prevalence, burden, diabetes-related foot disease, diabetic foot ulcer, diabetic foot infection, diabetes-related lower-extremity amputation, New South Wales, Australia, 45 and Up Study, linked data

## Abstract

Diabetes-related foot disease (DFD) is a major public health concern due to the higher risks of hospitalisation. However, estimates of the prevalence of DFD in the general population are not available in Australia. This study aims to estimate the prevalence of DFD and diabetes-related lower-extremity amputation (DLEA) among people aged 45 years and over in New South Wales (NSW), Australia. The NSW 45 and Up Study baseline survey data of 267,086 persons aged 45 years and over, linked with health services’ administrative data from 2006 to 2012 were used in our study. Of these, 28,210 individuals had been diagnosed with diabetes, and our study identified 3035 individuals with DFD. The prevalence of DFD, diabetic foot ulcer (DFU), diabetic foot infection (DFI), diabetic gangrene (DG), and DLEA were 10.8% (95%CI: 10.3, 11.2), 5.4% (95% CI: 5.1, 5.8), 5.2% (95%CI: 4.9, 5.5), 0.4% (95%CI: 0.3, 0.5), and 0.9% (95%CI: 0.7, 1.0), respectively. DFD, DFU, DFI, DG, and DLEA were the most common among those who were older, born in Australia, from low-income households (<AUD 20,000), or were without private health insurance. Interventional messages to reduce all forms of DFD should target those who are from high-risk groups.

## 1. Introduction

Diabetes-related foot disease (DFD) is a serious public health issue that results in a significant burden on healthcare resources and that severely impacts the quality of life of individuals [1,2]. Globally, about 83 to 148 million people with diabetes (19–34% of the total population with diabetes) are expected to develop a foot ulcer in their lifetime [3]. Half of these foot ulcers become infected [4], with over 15% requiring lower-extremity amputation [5]. DFD is responsible for around 5800 lower limb amputations in Australia annually [6], putting it at the top of the list among developed nations. These high rates have continued to be steady over the last decade [7] and have not improved with the country’s economic growth.

DFD is a major cause of hospitalisation worldwide, which is mainly due to the lower limb amputation resulting from the disease [3,8]. People with DFD have higher hospitalisation risks than patients with heart disease, kidney disease, or lung disease [9]. In Australia, people with DFD had the greatest number of hospital bed days, with a 26-day average stay in hospital due to lower limb amputation in 2004–2005 compared to other diabetes complications [7,10]. Clarke et al. (2008) reported that DFD had the highest length of inpatient hospital stays, with 48 and 57 days, respectively, for patients with diabetic foot ulcer (DFU) and lower limb amputation [11]. In comparison, studies by Comino et al. (2015) and the Australian Institute of Health and Welfare (2019) reported that patients with cardiovascular disease and heart failure stayed in the hospital for about 8.2 days and 3.4 days, respectively, while those with chronic obstructive airways disease had a length of hospital stay of approximately 2.7 days [12,13].

An excessive duration of stay at hospital places a significant strain on the healthcare system’s resources, leaving less room to use them elsewhere. It is five times more costly to provide health care to a person with DFU [14]. Both the hospital and non-hospital costs associated with amputation and foot ulcers were the second and third most expensive diabetes-related acute complications, respectively, in Australia in 2012 [7]. In 2017, DFD was estimated to cost the Australian health system AUD 1.6 billion annually, equating to about 0.9% of total health spending [15,16].

DFD and lower limb wounds are associated with significant morbidity and mortality. In 2016, DFD was ranked 11th in the global burden of disease, which was higher than ischemic stroke (17th), ischemic heart disease (29th), and chronic kidney disease due to diabetes (52nd) [17]. After amputation, the mortality rate varied between 39–80% over a five-year span, which was much higher than the mortality rate following most malignancies [18]. In 2005, the most recent year for which statistics are available, over 1000 deaths were attributable to DFD, accounting for about 8% of all diabetes-related deaths in Australia [19].

DFD, despite being a major health problem, is often overlooked in Australia [7]. There is a paucity of data on DFD, without which it is hard to understand and assess the extent of their burden [20]. Zhang et al. (2021) noted that very few studies in Australia reported the prevalence of different types of DFD [15,21,22,23]. The studies that have been conducted on DFD have predominantly used the inpatient diabetes population, specific ethnic groups, or geographic areas [15,22,23]. These studies could overestimate the prevalence burden, as they might represent people with diabetes who also have severe conditions [12]. This is supported by a systematic review conducted by Zhang et al. (2021) that cast doubt on the accuracy of the reported prevalence and highlighted the necessity to undertake a large epidemiological study involving a representative population.

The population of the state of New South Wales (NSW) is representative of the Australian population due to its diverse geography and demography [24]. The state has the highest proportion (35%) of people with diabetes as a proportion of the total Australian population with diabetes [24]. However, there are limited descriptive data on DFD prevalence by social characteristics in NSW.

This study will evaluate the prevalence of different types of DFD and related social and epidemiological characteristics using this large cohort study in NSW. The current paper has three key aims. First, the study will estimate the prevalence of DFD and its different types: diabetic foot ulcer (DFU), diabetic foot infection (DFI), diabetic gangrene (DG), DLEA, and other DFD. The second aim is to assess demographic, socioeconomic, lifestyle, and health-related characteristics among the study participants with diabetic foot complications. Third, the study will explore geographic variation in the prevalence of all DFD types and DLEA.

### Ethical Clearance

The current study has three ethics approvals: NSW Population and Health Services Research Ethics Committee Approval Number: HREC/13/CIPHS/8; Western Sydney University Human Research Ethics Committee Number: H12215; and ACT Health Human Research Ethics and Governance Approval Number: ETHLR.12.173.

## 2. Materials and Methods

### 2.1. Data

The study used data from five different sources that were linked by a unique person number. A description of each data source is provided in the below subsections.

#### 2.1.1. The 45 and Up Study Survey

Our study participants are based on the 45 and Up Study baseline survey, an ongoing study of the health and ageing of persons aged 45 years and over in NSW. The baseline survey was conducted from 2006 to 2009 [25,26]. About 267,153 individuals aged 45 years or over from NSW were recruited from a random sample of the Medicare (Australia’s public health insurance system) database, comprising 11% of the target population [27,28,29]. The process of recruiting this cohort and the sampling technique, including the sampling weights, have already been detailed elsewhere [27,30]. People who qualified for the study received an invitation with an information booklet, questionnaire, consent form, and reply-paid envelope. Participants enrolled in the study by completing the questionnaire and consent form and sending them back to the study coordination centre. The questionnaires were scanned electronically, and the data were double entered. The survey collected self-reported data on the participants’ sociodemographic, lifestyle, and health factors, including information on diabetes status, their age at first diagnosis of diabetes, presence of high blood pressure, high blood cholesterol, heart disease, stroke, asthma, and psychological stress [30].

#### 2.1.2. NSW Admitted Patient Data Collection (APDC)

The NSW APDC includes data on admissions into public hospitals, public mental hospitals, public multi-purpose services, private hospitals, and private day procedure centres [31]. International Classification of Diseases, 10th version, Australian Modification (ICD-10-AM) codes were used to classify the diagnoses in the APDC. In our research, the APDC data were accessible from 1999 to 2012. To determine a person’s diabetes status, our research used APDC data for the whole period available (1999–2021), while APDC records were retrieved from 2006 to 2012 to assess DFD.

#### 2.1.3. NSW Emergency Department Data Collection (EDDC)

The NSW EDDC includes data on emergency visits into public hospitals and in scope-contracted private hospitals in NSW [31]. International Classification of Diseases, 10th version, Australian Modification (ICD-10-AM) codes were used to classify the diagnoses in the EDDC. When the ICD-10 AM was not available, the EDDC recorded diagnoses using the International Classification of Diseases 9—Clinical Modification (ICD-9 CM) and the Systematized Nomenclature of Medicine—Clinical Terms (SNOMED- CT). In our study, EDDC data were available from 2005 to 2012. Our study utilised EDDC data from 2005 to 2012 to determine a person’s diabetes status. EDDC records were retrieved from 2006 to 2012 to assess DFD.

#### 2.1.4. Pharmaceutical Benefits Scheme Data

The Pharmaceutical Benefits Scheme Data (PBS) contain data on prescription medication claims that qualify for subsidy by the Australian government under the National Health Act 1953 [32,33]. In our study, PBS data were used to classify types of diabetes among survey participants. The PBS data utilised in our research spanned from 2004 to 2011.

#### 2.1.5. Medicare Benefits Scheme Data

The Medicare Benefits Scheme (MBS) dataset includes data on claims for medical and diagnostic services, the post codes of servicing provider’s practice location, the provider’s charge, and the benefit paid to patients under the Health Insurance Act 1973 [34,35]. Our study used postcode data to approximate the residence of the study participants. The postcode data used for this study were from six months before and after the recruitment of the 45 and Up Study participants. Therefore, the MBS data used in our study ranged from 2005 to 2010.

#### 2.1.6. Linkage of Records

The 45 and Up Study data were linked to the APDC and EDDC by the NSW Centre for Health Record Linkage (CHeReL) [36], whereas the MBS and PBS data were linked by the Sax Institute [37]. CHeReL and the Sax Institute used a probabilistic matching procedure to link the data. A unique person project number (PPN) was assigned to the linked records, and these records were then returned to the data custodians. The study team was provided with de-identified data uploaded to the Secure Unified Research Environment (SURE), a secure online workspace developed by the Sax Institute.

About 267,153 participants joined the baseline survey of the 45 and Up Study [28]. The Sax Institute supplied the data of 267,112 participants for our study. The difference in the cohort size is due to the ongoing maintenance and data cleaning by the Sax Institute [28]. A further 26 participants whose age was less than 45 years old or who had missing data were dropped from the survey. Therefore, in our study, the number of participants for which different data were available in all of the datasets, when using PPN, was 267,086. Out of 267,086 participants, this study identified 28,210 individuals with diabetes whose data were utilised for the analysis. Our study found 3035 persons with DFD during 2006–2012.

### 2.2. Identification of Diabetes and Its Types, and Different Types of DFD and DLEA

**Figure 1:**
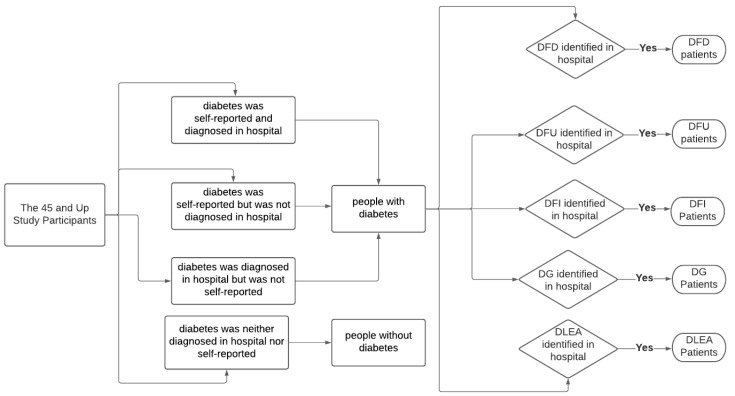
Identification of diabetes and different types of DFD and DLEA; DFD: diabetes-related foot disease, DFU: diabetic foot ulcer, DFI: diabetic foot infection, DG: diabetic gangrene, DLEA: diabetes-related lower extremity amputation.

The method of identifying individuals with diabetes and diabetic foot problems is shown in Figure 1. Different types of DFD and DLEA were identified in two steps. The first stage was to assess diabetes status using the 45 and Up Study survey data or hospital diagnosis codes [38]. The 45 and Up Study participants were asked: “Has a doctor ever told you that you have diabetes?” If they responded “yes,” they were assigned to have diabetes. Additionally, individuals were classified as having diabetes if they were diagnosed with diabetes during their hospitalisations or emergency department visits. In the second stage, the identification of different types of DFD and DLEA was only ascertained if the foot complications that were found concurrently with or after the diagnosis of diabetes (Figure 1). In this study, DFD included ulcer of a foot or lower limb, decubitus ulcer, peripheral angiopathy with or without gangrene, cellulitis of a toe or lower limb, osteomyelitis, mono/polyneuropathy of a lower limb, diabetic neuropathic arthropathy, and diabetes-related amputation of a lower limb [39,40,41,42]. DFU included ulcer of foot or lower limb, and decubitus ulcer, whereas DFI included cellulitis and osteomyelitis of toe or lower limb [23,39,43]. Peripheral angiopathy with gangrene and other gangrene of the lower extremities were classified as DG. Mononeuropathy, polyneuropathy, peripheral angiopathy without gangrene, diabetic neuropathic arthropathy, and other peripheral circulatory complications were included in other DFD [39,40,41,42]. Diagnostic codes of diabetes and related diabetic foot complications are shown in Appendix A Table A1 and Table A2.

**Figure 2:**
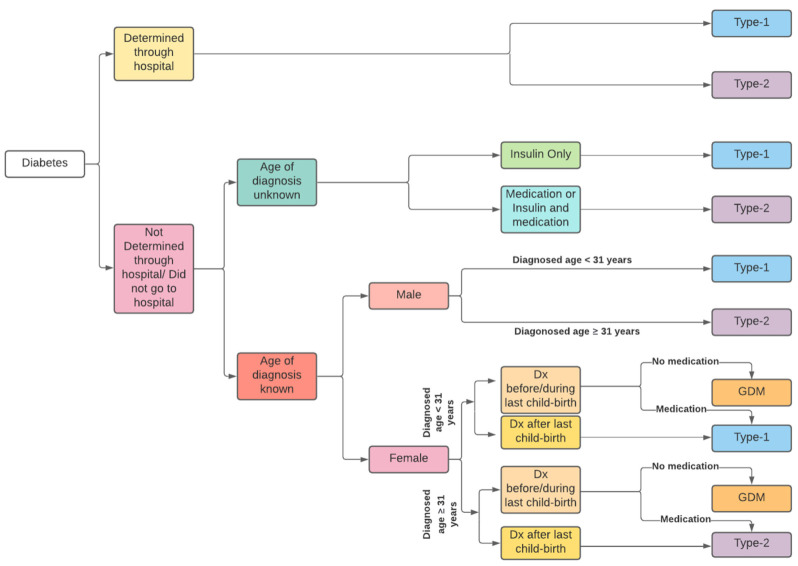
Identification of type of diabetes from the 45 and Up Study survey linked with health administrative data; Dx: diagnosed, GDM: gestational diabetes mellitus.

The conceptual framework presented in Figure 2 was followed to identify patients with different types of diabetes. This study divided individuals with diabetes into two categories based on hospital diagnosis codes: determined cases and undetermined cases. Individuals recorded with the same diabetes diagnosis codes across all hospital admissions during 1999–2012 were classified as determined cases. For the determined group, type 1 diabetes was assigned to patients with the E10 code, and type 2 diabetes was assigned to patients with the E11 code. Individuals whose diabetes types were not determined through the hospital—either for different diabetes diagnosis codes in different admissions or because they did not go to hospital during 1999–2012, were assigned as undetermined cases. Undetermined cases were classified by the strategies adopted in the study by Comino and her co-authors [44], as shown in Figure 2.

For the undetermined group, the ages of the individuals were compared to a cut-off point to identify the diabetes type in the presence of self-reported age of diabetes diagnosis. Following Comino et al. (2013), we hypothesised that if a male was diagnosed before the age of 31, he was more likely to have type 1 diabetes, and vice versa [44]. The cut-off age for females stayed the same. Yet, another assumption was based on the delivery of the patient’s last child. If the diagnosis was before the age of 31 and after the last childbirth, they were likely to have type 1 diabetes. Again, for females aged less than 31 years old, if the diagnosis was before or at the time of last childbirth and the usage of diabetes-related medicine occurred at later stages of life, then they were classified as diabetes type 1 patients. On the other hand, females satisfying the same condition but aged 31 years and above were classified as having type 2 diabetes.

Further investigation was conducted to classify types of diabetes among the participants belonging to undetermined cases whose age of diagnosis was unknown. The PBS dataset was used to identify the claims on insulin and oral hypoglycaemic agents for these individuals. If the individuals only made claims on insulin, then they were classified as type 1 diabetes patients. For other cases, type 2 diabetes was assigned.

### 2.3. Studied Variables

The prevalence of DFD, DFU, DG, DLEA, and other DFD were estimated across different factors during 2006–2012. This study divided these factors into four categories: demographic, socioeconomic, lifestyle risk factors, and other health status variables.

Gender, age, marital status, the remoteness of residence, country of birth, and whether they spoke a language other than English at home were among the demographic factors considered in this study. In contrast, the highest education level, socioeconomic status, household income, work status, and private health insurance status were the studied socioeconomic factors. The private health insurance variable was divided into three categories: “No” (without a Department of Veterans’ Affairs (DVA) card or a concession card), “No” (with a DVA card or a concession card), and “Yes”. The DVA card is available to current or former members of the Australian Defence Force and their families, while the concession card is available to eligible individuals. Many factors influence concession card eligibility, including age, income, and disability status. Compared to non-holders, DVA or concession card holders may receive higher discounted health services and medication [45,46].

Self-reported smoking status, alcohol consumption, physical activity, and fruit and vegetable intake were among the lifestyle risk factors in this study and were classified following earlier Australian research [47,48,49]. Smoking status was classified into two categories: never being a regular smoker or ever being a regular smoker [47]. Alcohol consumption was measured based on the self-reported number of drinks per week and was categorised as more than 14 drinks per week and 14 drinks or less per week [48]. Total weekly physical activity time was categorized using the cut-off points of 150 min and 300 min [49]. Adequate fruit consumption was defined as two or more servings per day, and adequate vegetable consumption was defined as five or more servings per day [48].

Variables related to health status were types of diabetes, diabetes duration, body mass index (BMI), and the presence of comorbidities BMI was calculated using self-reported height and weight according to the National Health and Medical Research Council guidelines [50]. Presence of different comorbidities such as high blood pressure, high blood cholesterol, heart disease, stroke, and asthma were ascertained if participants were told by the doctor that they had these conditions. The Kessler-10 (K10) scale was used to categorise psychological distress into three groups: “low”, “moderate”, and “high/very high” [51].

### 2.4. Statistical Analysis and Spatial Analysis

The distribution of all types of DFD was presented as a percentage, and prevalence analyses were performed using the Stata statistical software package version 16.1 (Stata Corp., College Station, TX, USA) with “*svy*” commands that allow for adjustments of the sampling weights in the survey. The Taylor series linearisation method was applied in the survey when estimating their 95% confidence intervals (CI) around prevalence estimates. Chi-squared tests were used to test the significance of associations. If the variable had more than two categories, then the non-overlapping CIs of different categories of the same variable indicated that the groups were significantly different from each other.

The prevalence of different types of DFD and DLEA were shown in heat maps according to the local health district (LHD) of NSW. In Australia, LHDs are considered as statutory corporations that are responsible for administering public hospitals and health facilities as well as for delivering health services to defined geographic regions across the state [52]. To promote, protect, and maintain the health of the community, it is imperative to have an understanding of the prevalence burden of the disease at the local level. With the objective of informing decision makers about the geographic disparity of the prevalence of DFD, a spatial analysis was conducted.

The spatial analysis was performed in several steps for the participants with diabetes. First, the 45 and Up study data were merged with the MBS data. Second, MBS data on the postcodes of the individuals’ residences were kept from within six months (before and after) from the date of the survey. Third, individuals with residence postcodes were assigned to the postcodes that were the most frequent. Fourth, using R version 3.6 software, postcodes were mapped into LHD utilising associated shapefiles. Fifth, the merged 45 and Up Study and MBS data were combined with the APDC and EDDC data to retrieve information on their diabetic foot complications. Finally, the age-standardised prevalence of DFD and its various forms and DLEA were computed and shown as heat maps.

## 3. Results

The study identified 3035 individuals with DFD among 28,210 people with diabetes who were aged 45 years and over residing in NSW during 2006–2012. The distribution of patients with DFD is reported in Table 1. Among the 3035 people with DFD, 2080 individuals experienced one type of DFD, and the rest had multiple foot complications. Table 1 illustrates that almost half of the patients with DFD developed foot ulcer and foot infection. About 4% of individuals with DFD suffered from gangrene. The number of people who had DLEA was 222, more than 7% of the diabetic foot patients. Among other consequences, about 39% of individuals had other types of DFD, including polyneuropathy and mononeuropathy, peripheral angiopathy without gangrene, diabetic neuropathic arthropathy, and other peripheral circulatory complications.

The prevalence of different types of DFD and DLEA among population with diabetes in terms of social factors are documented in Table 2 and Table 3. The characteristics of survey participants with diabetes and unweighted prevalence are also reported in Appendix A Table A4 and Table A5, respectively. DFD prevalence for people aged 45 years and over and residing in NSW was found in about 11% among the people with diabetes during 2006–2012 (Table 2). During the same period, the prevalence of amputation (below/above knee, foot, or toe) was calculated as 0.9% (Table 2). The estimated prevalence of foot ulcers and foot infection suggests that around 5% of people with diabetes had these foot complications, whereas about 4% had other types of DFD (Table 2). The prevalence of different types of DFD and DLEA according to demographic, socioeconomic, lifestyle, and health-related factors are discussed in the following paragraphs.

The prevalence of all types of DFD and DLEA are provided in Table 2 in terms of different demographic factors. Overall, DFD was the most prevalent among the group aged 75 years above (19%), which was followed by the 65–74 years age group (10%). The oldest age category (75 years and above) was also to be the predominant group for all types of DFD and DLEA. The prevalence of all DFD and DLEA was higher in males than it was in females, with the exception of DFU and DFI. All forms of DFD, except for DG, were found to be more common among widowed individuals. People from remote/very remote areas had the highest prevalence of DFD (15%), with DFI and DG being equally prominent. With the exception of DG, all of the different kinds of DFD and DLEA were more common among individuals born in Australia than those born elsewhere (12% vs. 9%). People who exclusively spoke English at home suffered s from DFU, DFI, DLEA, and other DFD in higher proportion compared to those who could communicate in other languages.

Table 2 also illustrates the estimates of prevalence of DFD, DFU, DFI, DG, and DLEA in terms of several socioeconomic factors: highest education level, Socioeconomic Indexes for Areas (SEIFA), annual household income, work status, and private health insurance. The prevalence of all diabetic foot complications was the highest among those without a school certificate. People from the lower-income category (less than AUD 20,000) had the highest prevalence of DFU (7%), DFI (7%), DG (0.7%), DLEA (1%), and other DFD (5%). DFU, DFI, DLEA, and other DFD were found to be the most prevalent among individuals who had already retired from the workforce. Diabetic foot complications were the least predominant in people with private health insurance.

The prevalence of DFU, DFI, and other DFD varied significantly across various lifestyle variables, including smoking and physical activity (Table 2). Individuals who smoked regularly at any point in their lives showed a greater prevalence of overall DFD than those who never smoked regularly (12% vs. 10%). People who engaged in less moderate-to-vigorous physical activity (less than 150 min of moderate-to-vigorous physical activity per week) had a higher prevalence of DFD (15%), DFU (8%), DFI (7%), and DLEA (over 1%) (Table 2).

**Figure 3:**
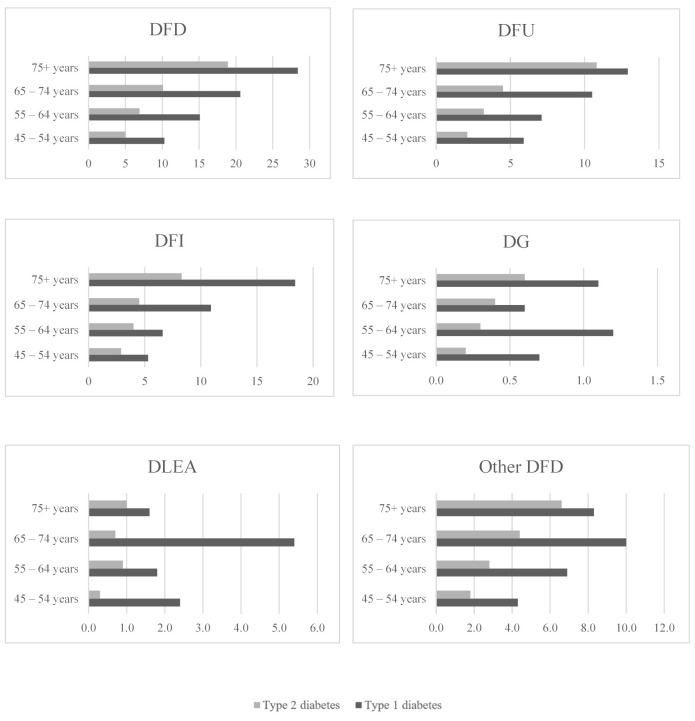
Prevalence of diabetic foot complications by age groups and type of diabetes.

Prevalence of all types of DFD and DLEA according to different health status factors are provided in Table 3. Compared to people with type 2 diabetes, the overall DFD was more common in type 1 diabetes patients (16% vs. 11%), including DFU, DFI, DG, DLEA, and other DFD. Further analysis suggests that DFU, DFI, and DG were substantially high among people with type 1 diabetes aged over 75 years of age (Figure 3). However, DLEA and other DFD were predominant among people with type 1 diabetes aged 65–74 years (Figure 3).

People who had diabetes for 15 years or more had the highest overall prevalence of DFD (17%). The DLEA prevalence was more than double (2%) for this group compared to the overall prevalence of DLEA (0.9%). Again, people who had had diabetes for 15 years or more had the highest prevalence of DFU (10%), DFI (8%), DG (0.8%), and other DFD (8%). Individuals with a BMI less than 25 (underweight/normal) had a 20% prevalence of overall DFD. DFU and DFI were also more often diagnosed in normal/underweight individuals with diabetes.

The overall DFD prevalence was more common in individuals with cardiovascular disease (14%), stroke (17%), asthma (13%), and high/very high psychological distress (13%) than it was in those without these conditions. The prevalence of DFU significantly varied between different groups of cardiovascular disease, stroke, asthma, and psychological distress. Individuals with these comorbidities comprised the majority of those diagnosed with DFU. Again, these conditions, with the exception of asthma, were all associated with a higher prevalence of DFI, while DLEA was only found to be significantly more prevalent in individuals with cardiovascular problems among the comorbidities.

**Figure 4:**
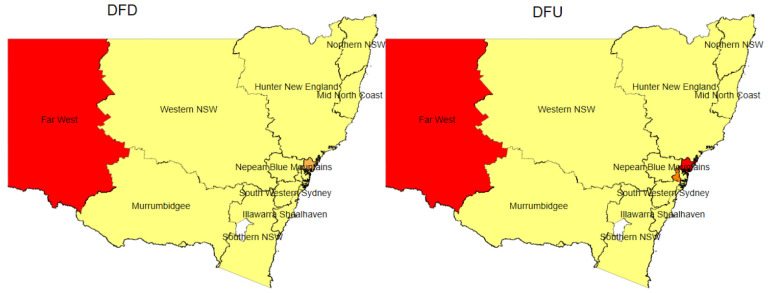

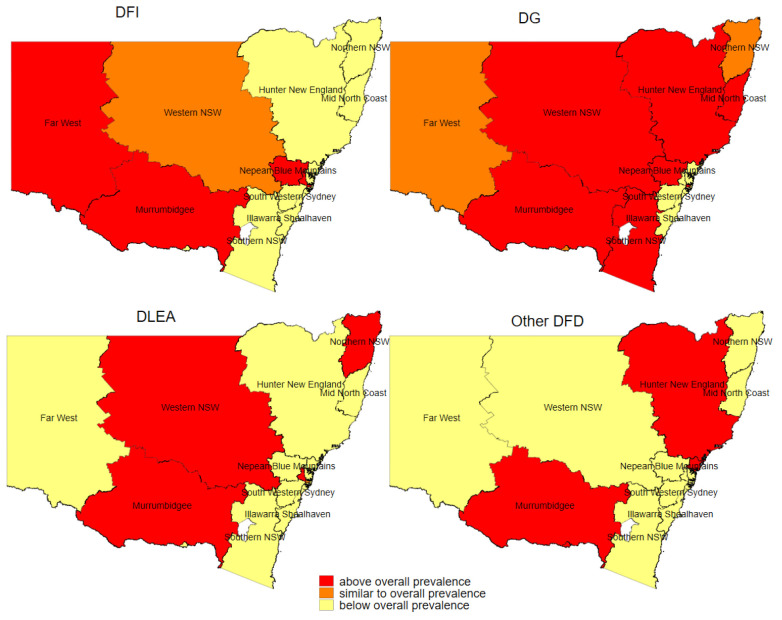
Age-standardised prevalence of diabetic foot complications in different local health districts (LHDs) of the state of New South Wales (NSW) among individuals aged 45 years and over during 2006–2012.

The age-standardised prevalence of diabetic foot complications in different LHDs of NSW state among individuals with diabetes aged 45 years and over during 2006–2012 is presented in Figure 4. Data on the prevalence estimates are shown in Appendix A Table A6. Figure 4 depicts that Far West was an LHD of concern for DFU and DFI, having higher prevalence than the overall prevalence. In addition, Central Coast and Nepean Blue Mountains had higher proportions of DFU and DFI, respectively. Murrumbidgee LHD had a higher prevalence of DFI, DG, DLEA, and other DFD. Northern and Western NSW were amongst the other LHDs that had an above average prevalence of DLEA.

## 4. Discussion

Our study set out to describe the prevalence burden of different types of DFD in NSW, Australia, among individuals with diabetes aged 45 years and over during 2006–2012 and to identify those who are at risk for these conditions. The study found that the overall prevalence of DFD was 10.8% among individuals with diabetes. The prevalence of DFU, DFI, DG, DLEA, and other DFD were estimated as 5.4%, 5.2%, 0.4%, 0.9%, and 4.1%, respectively. The study highlighted that DFU, DFI, and DLEA were found to be the most prevalent among individuals who were older, born in Australia, widowed, were from low-income households, and did not have private health insurance. The burden of DFD was borne in a higher proportion by those who had had diabetes for over 15 years, who had type-1 diabetes, and who had high psychological distress.

The literature on the prevalence of DFD varies considerably in study design, time-period, definition of the numerator and denominator population, and population demographics. As a result, the findings regarding the prevalence of DFD are mixed. According to the Fremantle Study (a longitudinal community based-study conducted among people with diabetes living around Fremantle Hospital catchment area of Western Australia) [22] and the Australian Diabetes, Obesity, and Lifestyle (AusDiab) Study (a cross sectional population-based survey of adults aged 25 years and above residing in randomly selected urban and rural areas of Australia during 1999–2000) [38], the prevalence of DFU in the population with diabetes in Australia varied between 1.2% and 2.5%. These estimates were lower compared to global pooled prevalence (4.6%) [53]. However, the most recent study—conducted in the Queensland state of Australia (2013) by Lazzarini et al. (2016)—reported that the DFU prevalence among diabetes inpatients to be 15.1% [15]. This is compared to a global-pooled prevalence of 7.1% among the inpatient population with diabetes [53]. In our study, the prevalence of DFU among the people with diabetes was found as 5.4%, which translates into 7.5% prevalence among inpatient population with diabetes (Appendix A Table A3). Therefore, similar to the Lazzarini et al. (2016), in inpatient-based research [15], the prevalence of DFU was higher than the global prevalence among the general population (5.4% vs. 4.6%) and population with diabetes (7.5% vs. 7.1%). The variation in the prevalence of DFU in the Australian setting, including this study, may be ascribed, in part, to differences in population age, demographics, and time period.

Our study found that individuals who were married or in a de-facto relationship had a lower prevalence of DFD. A possible explanation for this could be that people who were living with their partners were more likely to identify any adverse foot condition sooner with help from their partner that might have gone unnoticed otherwise. Although not highlighted and explained, the study by Abbott et al. corroborates our finding [54].

Our study found that the prevalence of different DFD and DLEA was higher among individuals with low socioeconomic status. For instance, the prevalence in the lowest income group was three times higher than it was in the highest income group. This is not unexpected since income impacts one’s capacity to obtain various healthcare services, including those that are necessary for diabetic foot problems and preventative measures. However, at the same time, it is important to note that Australians have access to a publicly funded unlimited general practice (GP) service with a maximum of 5 visits to be eligible for allied health services annually, which includes podiatry services [55]. GPs cannot claim reimbursement for wound dressings, which may mean that the care may not be optimal. Patients with DFD, on the other hand, need urgent and unrestricted access to comprehensive foot care, which includes specialist services [56]. Specialist services, public podiatry services beyond the annual limit or private podiatry services, and preventive measures, such as buying special shoes or stockings, incur out-of-pocket costs. Therefore, low income may adversely impact access to these healthcare services and goods.

One concern for the income variable in our study is 25% missing values in the sample of interest. Further analysis revealed that missing values were evenly distributed among individuals with and without private health insurance. Private health insurance can be a proxy for high socioeconomic status [57]. Our data also supported this claim, as the percentage of people with health insurance among the highest income group was 83% compared to 29% in the lowest income group. Therefore, it can be said that income data were missing at random. The prevalence estimate can still be biased, even if data are missing at random [58]. Nonetheless, the low prevalence of different diabetic foot complications among individuals with private health insurance—a strong predictor of high income or socioeconomic status—reconfirms the claim on the income and the prevalence of diabetic foot complications. Previous studies also corroborate our findings [59,60]. Apart from this, the low prevalence of DFD among private health insurance users may be attributed to the greater utilisation of podiatrist services and those for better foot health management. Individuals with private health insurance may have their podiatry and customized footwear expenses reimbursed by their insurer, while those without private health insurance do not have such options. Those without private health insurance may not obtain podiatrist treatments due to financial constraints [61].

The prevalence of DFD was reported to be higher among individuals with high psychological distress in our study. An understanding of psychological issues are important factors along with biological and physiological aspects to provide holistic care to patients with DFD [62]. Although the analysis of the pathophysiological pathway is beyond the scope of our study, a plausible explanation could be that individuals with a very high level of psychological distress can compromise their compliance on self-management (including glycaemic control) and consequently on wound healing. Our finding is consistent with previous studies [63,64] although there are studies that contradict the finding [65,66].

Our study identified LHDs of concern for several diabetic foot problems, including Far West, Murrumbidgee, Northern NSW, Western NSW, Central Coast, and Nepean Blue Mountains. One explanation could be that these LHDs belong to rural and regional parts of NSW, which has inadequate access to podiatry services. The Australian Institute of Health and Welfare (AIHW) reported that there were about 6.6 full-time equivalent (FTE) podiatrists per 100,000 people in remote areas of NSW in contrast to 12.9 FTE podiatrists per 100,000 in the metropolitan NSW. A study by Common et al. also suggested that the lack of podiatrists might be responsible for decreased capacity in the primary detection and prevention of DFD [23]. Further analysis revealed that the proportion of people with low socioeconomic status and without private health insurance were prominent in these regions. As a result, financial constraints may limit their access to private podiatry services and specialist services [67]. Another plausible explanation could be that people may not be comfortable accessing healthcare far from their residence in remote areas [67]. Therefore, both low access and low affordability of healthcare could be the main bottlenecks for people with diabetes in these regions to avail the necessary multidisciplinary foot healthcare to reduce the risk of foot complications.

One of the strengths of the current study is the linkage of the survey data with health utilisation data to identify people with diabetes and thus diabetic foot complications. The Australian Bureau of Statistics (ABS) 2011–2012 Health Survey report showed that self-reported diabetes status might underestimate people with diabetes by 20% [19]. Again, hospital coding is not always accurate in detecting diabetes. Since there is no separate classification to identify diabetic foot complications from hospital diagnosis codes, combining information on diabetes status from survey and hospital data could improve the identification of diabetes and diabetic foot complications. Another strength of our research is that it could demonstrate its findings using a representative general population, this distinguishes it from previous studies that were based on hospitalised patients or individuals from a certain region or ethnicity [40,68,69,70,71].

Our study has a few limitations. First, if the diabetic foot complications were input incorrectly or not input at all during the admission [69], then the estimated DFD and its various types would be underestimated. However, previous research found that the coding precision during the admission was adequate for making accurate estimations [41]. Second, our study did not distinguish between major and minor amputations. Third our study could not portray the prevalence of diabetic foot complications by indigenous status due to not having the approval for that information. Fourth, data on household income contained missing information. However, the findings remained the same when an alternative socioeconomic indicator was used. Fifth, our study did not have any measure of glycaemia, which might underestimate diabetes. This issue was mitigated to a large extent by the usage of linked data. Sixth, types of diabetes can be subjected to assumptions made in this study. Lastly, time lags are inherently related to large data linkage-based studies due to the time involved in obtaining, training, linking, cleaning, and validating the cross-system data linkage. Despite these drawbacks, the current study’s use of a large representative survey in conjunction with administrative health data to understand the prevalence burden of diabetic foot complications in the older Australian population is a significant strength.

## 5. Conclusions

This study has offered a reference to understand the prevalence burden of diabetic foot complications in an Australian population aged 45 years and above. The study found that over 1 in 10 people aged 45 years and older with diabetes had DFD in NSW and tailored interventional messages to reduce all forms of diabetes-related foot diseases, which should target all high-risk groups, especially those from lower socioeconomic status. In this regard, the critical role of a multidisciplinary team, including a GP, nurse, podiatrist, other allied health professional, and an endocrinologist, is undeniable for the management and treatment of patients with DFD. This may reduce the risk of the hospitalisation of individuals with DFD and thereby reduce the economic burden due to DFD. The findings of our study may help in comprehending the magnitude of the need for health professionals to manage and treat diabetic foot disease in the NSW region. For policy makers, the findings from this study can also be utilised to develop preventive and treatment strategies to tackle DFD, enhance the overall quality of life, and decrease the large hospitalisation burden. The results of this research are broadly relevant in an Australian setting, and data on the prevalence of DFD and DLEA are of interest to the international diabetic foot community. Future studies involving the analysis of the economic impact of the DFD on population health can complement the findings of this study to identify strategies to reduce the burden of DFD. The study also warrants the necessity of separate diagnostic codes for DFD in the existing disease classification system in order to capture the disease more precisely.

## Figures and Tables

**Table 1:** Distribution of patients with diabetic foot complications (*n* = 3035).

Diabetes-Related Foot Disease (DFD) (*n* = 3035) ^a^	Frequency	Percentage
Diabetic foot ulcer (DFU)	1503	49.5
Diabetic foot infection (DFI) ^b^	1448	47.7
Cellulitis	1335	44.0
Osteomyelitis	219	7.2
Diabetic gangrene (DG)	123	4.1
Diabetes-related lower limb amputation (DLEA) ^c^	222	7.3
Toe	184	6.1
Foot	14	0.5
Below knee	34	1.1
Above knee	33	1.1
Other diabetes-related foot disease (Other DFD) ^d^	1189	39.2
Peripheral angiopathy without gangrene	670	22.1
Mononeuropathy	332	10.9
Polyneuropathy	270	8.9
Diabetic Neuropathic arthropathy	32	1.1
Other peripheral circulatory complication	47	1.6

Note: ^a^ Among 3035 individuals with DFD, 2080 individuals (68.5%) were diagnosed with one type of foot complication, 602 individuals (20%) were diagnosed with two types of foot complications, 227 (7.5%) had three types of complications, whereas 126 (4%) had four or more types of foot complications. ^b^ Among 1448 patients with DFI, 1229 patients had cellulitis only, and 113 had osteomyelitis and 106 had both types of infections. ^c^ Among 222 individuals with DLEA, 184 had one type of DLEA, whereas 38 patients had multiple types of DLEA. ^d^ Among 1189 patients with other types of DFD, 605 patients had one type of other DFD, and 584 patients had multiple types of other DFD.

**Table 2:** Prevalence of DFD, DFU, DFI, DG, DLEA, and other DFD among people with diabetes in terms of demographic, socioeconomic and lifestyle risk factors.

	Diabetes-Related Foot Disease (DFD) (*n* = 3035)	Diabetic Foot Ulcer (DFU) (*n* = 1503)	Diabetic Foot Infection (DFI) (*n* = 1448)	Diabetic Gangrene (DG) (*n* = 123)	Diabetes-Related Lower Extremity Amputation (DLEA) (*n* = 222)	Other Diabetes-Related Foot Disease (Other DFD) (*n* = 1189)
	Prevalence (95% CI)	Prevalence (95% CI)	Prevalence (95% CI)	Prevalence (95% CI)	Prevalence (95% CI)	Prevalence (95% CI)
Overall	10.8 (10.3, 11.2)	5.4 (5.1, 5.8)	5.2 (4.9, 5.5)	0.4 (0.3, 0.5)	0.9 (0.7, 1.0)	4.1 (3.8, 4.5)
						
**Demographic factors**						
Age ^a^						
45–54 years	5.4 (4.5, 6.4) ***	2.4 (1.8, 3.1) ***	3.1 (2.5, 3.9) ***	0.2 (0.1, 0.5)	0.4 (0.3, 0.8)	1.7 (1.3, 2.2) ***
55–64 years	7.2 (6.5, 7.9)	3.3 (2.8, 3.9)	4.1 (3.6, 4.7)	0.4 (0.2, 0.7)	1.0 (0.7, 1.4)	3.0 (2.5, 3.5)
65–74 years	10.3 (9.5, 11.1)	4.6 (4.1, 5.2)	4.7 (4.1, 5.2)	0.4 (0.3, 0.6)	0.8 (0.6, 1.1)	4.5 (4.0, 5.1)
75+ years	19.0 (18.0, 20.1)	10.8 (10.0, 11.7)	8.5 (7.7, 9.3)	0.6 (0.5, 0.9)	1.1 (0.8, 1.4)	6.5 (5.9, 7.2)
Gender						
Male	11.5 (10.9, 12.1) ***	5.7 (5.3, 6.2)	5.4 (5.0, 5.9)	0.6 (0.5, 0.8) ***	1.1 (0.9, 1.3) ***	4.7 (4.4, 5.2) ***
Female	10.0 (9.3, 10.7)	5.1 (4.6, 5.6)	4.9 (4.5, 5.5)	0.2 (0.1, 0.3)	0.6 (0.4, 0.8)	3.3 (2.9, 3.7)
Current marital status ^a^						
Single	12.2 (10.5, 14.1) ***	7.1 (5.9, 8.6) ***	7.9 (6.5, 9.7) ***	0.8 (0.4, 1.4)	1.2 (0.8, 1.9) ***	3.7 (2.9, 4.8) ***
Married/defacto	8.8 (8.4, 9.3)	4.1 (3.8, 4.5)	4.0 (3.7, 4.3)	0.4 (0.3, 0.5)	0.7 (0.5, 0.8)	3.6 (3.3, 3.9)
Widowed	16.4 (15.1, 17.9)	9.0 (8.0, 10.2)	8.2 (7.2, 9.3)	0.4 (0.3, 0.7)	0.9 (0.6, 1.3)	5.3 (4.6, 6.1)
Divorced/separated	11.7 (10.4, 13.2)	5.9 (4.9, 7.0)	5.7 (4.7, 6.8)	0.6 (0.3, 1.2)	1.4 (0.9, 2.2)	4.7 (3.9, 5.7)
Remoteness ^+,a^						
Major Cities	10.4 (9.8, 11.0) ***	5.4 (4.9, 5.9)	5.0 (4.5, 5.4) **	0.4 (0.3, 0.6) *	0.9 (0.7, 1.1)	4.0 (3.6, 4.4)
Regional	11.3 (10.7, 11.9)	5.4 (5.0, 5.9)	5.5 (5.1, 5.9)	0.4 (0.3, 0.6)	0.8 (0.7, 1.0)	4.5 (4.1, 4.9)
Remote/Very remote	15.4 (11.9, 19.7)	7.4 (5.1, 10.7)	8.6 (6.0, 12.2)	1.3 (0.5, 3.0)	1.3 (0.6, 3.1)	4.4 (2.7, 7.1)
Born in Australia ^b^						
Yes	11.8 (11.3, 12.4) ***	6.1 (5.7, 6.6) ***	5.9 (5.4, 6.3) ***	0.5 (0.4, 0.6)	1.1 (0.9, 1.3) ***	4.3 (4.0, 4.7) *
No	8.6 (7.9, 9.4)	4.1 (3.6, 4.6)	3.9 (3.5, 4.5)	0.3 (0.2, 0.5)	0.4 (0.3, 0.6)	3.5 (3.1, 4.1)
Language spoken other than English						
Yes	7.6 (6.7, 8.6) ***	3.6 (3.0, 4.3) ***	3.6 (3.0, 4.4) ***	0.3 (0.2, 0.6)	0.4 (0.3, 0.7) **	2.8 (2.3, 3.5) ***
No	11.6 (11.1, 12.1)	5.9 (5.5, 6.3)	5.6 (5.2, 6.0)	0.5 (0.3, 0.6)	1.0 (0.8, 1.2)	4.4 (4.1, 4.7)
**Socioeconomic factors**						
Education ^b^						
Less than high school	12.5 (11.8, 13.3) ***	6.4 (5.8, 7.0) ***	6.3 (5.8, 6.9) ***	0.5 (0.4, 0.8) *	1.1 (0.9, 1.4) **	4.5 (4.0, 5.0) ***
High school certificate/trade	10.7 (9.8, 11.6)	5.8 (5.1, 6.5)	4.4 (3.8, 5.0)	0.4 (0.2, 0.6)	1.0 (0.7, 1.3)	4.7 (4.2, 5.4)
Certificate/diploma	8.9 (8.0, 9.9)	4.0 (3.4, 4.7)	4.6 (3.9, 5.4)	0.3 (0.2, 0.5)	0.5 (0.3, 0.8)	3.4 (2.9, 4.1)
University degree or higher	7.0 (6.2, 8.0)	3.0 (2.4, 3.6)	3.4 (2.8, 4.1)	0.2 (0.1, 0.3)	0.5 (0.2, 0.9)	2.5 (2.0, 3.1)
SEIFA (IRSD) ^^,a^						
quantile 1 (least disadvantaged)	10.3 (9.4, 11.3)	5.2 (4.5, 5.9)	5.4 (4.7, 6.2)	0.4 (0.2, 0.6)	1.0 (0.7, 1.4)	3.5 (3.0, 4.2)
quantile 2	11.4 (10.4, 12.5)	5.9 (5.1, 6.7)	5.3 (4.6, 6.1)	0.5 (0.3, 0.8)	0.7 (0.5, 1.0)	4.3 (3.7, 5.0)
quantile 3	11.1 (10.1, 12.1)	5.7 (5.0, 6.6)	5.4 (4.7, 6.2)	0.4 (0.2, 0.9)	1.1 (0.8, 1.6)	4.7 (4.1, 5.4)
quantile 4	10.4 (9.5, 11.4)	5.1 (4.4, 5.9)	4.3 (3.7, 5.0)	0.4 (0.3, 0.7)	0.8 (0.5, 1.1)	4.1 (3.5, 4.8)
quantile 5 (most disadvantaged)	10.3 (9.4, 11.3)	5.2 (4.5, 5.9)	5.4 (4.7, 6.2)	0.4 (0.2, 0.6)	1.0 (0.7, 1.4)	3.5 (3.0, 4.2)
Annual household income (in AUD) ^c^						
<20 K	13.3 (12.5, 14.2) ***	6.8 (6.2, 7.4) ***	6.5 (5.9, 7.2) ***	0.7 (0.5, 1.0) ***	1.2 (0.9, 1.6) ***	5.0 (4.5, 5.5) ***
20 K–<50 K	9.4 (8.6, 10.3)	4.7 (4.1, 5.4)	4.2 (3.7, 4.8)	0.3 (0.2, 0.5)	0.6 (0.4, 0.9)	3.7 (3.2, 4.3)
>50 K	5.3 (4.6, 6.0)	2.4 (2.0, 2.9)	2.8 (2.4, 3.4)	0.1 (0.0, 0.2)	0.4 (0.2, 0.7)	1.5 (1.2, 1.9)
did not disclose	10.6 (9.5, 11.7)	5.7 (4.9, 6.6)	4.9 (4.2, 5.7)	0.5 (0.3, 0.8)	0.8 (0.5, 1.1)	4.0 (3.4, 4.7)
Work Status ^^^,b^						
Paid	4.6 (4.1, 5.2) ***	1.8 (1.4, 2.2) ***	2.6 (2.2, 3.1) ***	0.2 (0.1, 0.5)	0.5 (0.3, 0.8) *	1.6 (1.3, 2.0) ***
Retired	13.9 (13.2, 14.6)	7.3 (6.8, 7.9)	6.4 (5.9, 6.9)	0.5 (0.4, 0.6)	1.0 (0.8, 1.2)	5.3 (4.9, 5.8)
Other	11.8 (10.6, 13.1)	5.9 (5.0, 6.8)	5.9 (5.1, 6.9)	0.6 (0.4, 0.9)	1.1 (0.7, 1.5)	4.9 (4.1, 5.8)
Private Health insurance						
Yes	8.6 (8.0, 9.1) ***	4.2 (3.8, 4.6) ***	3.9 (3.5, 4.3) ***	0.3 (0.2, 0.4) *	0.5 (0.4, 0.7) ***	3.1 (2.8, 3.4) ***
No (without DVA/concession card) ^^^^^	10.2 (9.1, 11.3)	4.9 (4.1, 5.7)	5.5 (4.7, 6.4)	0.5 (0.3, 1.1)	0.8 (0.5, 1.3)	3.9 (3.2, 4.7)
No (with DVA/concession card)	14.1 (13.2, 15.0)	7.4 (6.8, 8.1)	6.9 (6.3, 7.5)	0.6 (0.4, 0.8)	1.4 (1.1, 1.7)	5.5 (5.0, 6.1)
**Lifestyle risk factors**						
Ever being a regular smoker ^a^						
Yes	12.1 (11.5, 12.8) ***	6.0 (5.5, 6.5) ***	5.6 (5.1, 6.1) **	0.5 (0.4, 0.7) *	0.9 (0.7, 1.2)	5.0 (4.6, 5.5) ***
No	9.4 (8.9, 10.0)	4.9 (4.4, 5.3)	4.8 (4.4, 5.3)	0.3 (0.2, 0.4)	0.8 (0.6, 1.0)	3.2 (2.8, 3.5)
Alcohol consumption ^b^						
≤14 drinks per week	10.9 (10.4, 11.3)	5.5 (5.1, 5.8)	5.2 (4.9, 5.6)	0.4 (0.3, 0.5)	0.8 (0.7, 1.0)	4.1 (3.8, 4.4)
>14 drinks per week	9.9 (8.7, 11.2)	4.8 (4.0, 5.8)	4.7 (3.9, 5.7)	0.6 (0.4, 1.0)	1.1 (0.7, 1.7)	4.2 (3.4, 5.0)
Total moderate-to-vigorous physical activity per week (minutes)						
<150 min	15.1 (14.3, 16.0) ***	8.2 (7.6, 8.9) ***	7.3 (6.7, 7.9) ***	0.6 (0.4, 0.7)	1.2 (1.0, 1.5) **	5.7 (5.2, 6.2) ***
150–300 min	8.6 (7.6, 9.6)	4.2 (3.5, 5.0)	4.0 (3.4, 4.7)	0.4 (0.2, 0.6)	0.6 (0.4, 1.0)	3.5 (2.9, 4.2)
>300 min	7.9 (7.3, 8.5)	3.5 (3.2, 3.9)	3.9 (3.5, 4.3)	0.3 (0.2, 0.5)	0.7 (0.5, 0.9)	2.9 (2.6, 3.3)
Vegetables intake ^b^						
<5 serves per day	10.5 (10.0, 11.1)	5.3 (4.9, 5.7)	4.9 (4.6, 5.3)	0.5 (0.4, 0.6)	0.8 (0.6, 1.0)	4.1 (3.7, 4.4)
5 or more serves per day	10.6 (9.9, 11.4)	5.3 (4.8, 5.9)	5.3 (4.8, 5.9)	0.3 (0.2, 0.5)	0.8 (0.6, 1.1)	3.9 (3.5, 4.4)
Fruit intake ^b^						
<2 serves per day	11.1 (10.4, 11.8)	5.7 (5.2, 6.3)	5.3 (4.8, 5.8)	0.5 (0.4, 0.7)	1.1 (0.8, 1.4) *	4.1 (3.7, 4.6)
2 or more serves per day	10.6 (10.0, 11.2)	5.2 (4.8, 5.7)	5.1 (4.7, 5.5)	0.4 (0.3, 0.5)	0.7 (0.6, 0.9)	4.0 (3.7, 4.4)

Notes: ***, **, and * denote *p*-values of 0.001, 0.01, and 0.05, respectively, for Pearson’s Chi-square test. Overlapping 95%CI indicated statistically non-significant. ^a^ Less than 1% missing values for age, marital status, remoteness, SEIFA, and ever being a smoker. ^b^ Less than 4% missing values for born in Australia, work status, fruit intake, vegetable intake, and alcohol consumption. ^c^ Annual household income has 25% missing values, including “did not disclose”. ^+^ Remoteness of residence is derived from the accessibility-Remoteness Index of Australia scores from 2006 (Australian Government Department of Health). These scores were calculated based upon distance by road to the nearest population centre where services could be obtained. The Australian Bureau of Statistics uses this score to classify different regions into major cities, inner regional, outer regional, remote, and very remote. ^^^ Socioeconomic status was assessed by index of relative socioeconomic disadvantage (IRSD), which is one of indices of Socioeconomic Indexes for Areas (SEIFA) (Australian Bureau of Statistics). ^^^^ “Other” category in work status included individuals who were sick, disabled, students, or unemployed. ^^^^^ DVA denotes Department of Veterans’ Affairs. DVA or concession card holders may receive higher discounted health services and medication.

**Table 3:** Prevalence of DFD, DFU, DFI, DG, DLEA, and other DFD among people with diabetes according to health status factors.

	Diabetes-Related Foot Disease (DFD) (*n* = 3035)	Diabetic Foot Ulcer (DFU) (*n* = 1503)	Diabetic Foot Infection (DFI) (*n* = 1448)	Diabetic Gangrene (DG) (*n* = 123)	Diabetes-Related Lower Extremity Amputation (DLEA) (*n* = 222)	Other Diabetes-Related Foot Disease (Other DFD) (*n* = 1189)
	Prevalence (95% CI)	Prevalence (95% CI)	Prevalence (95% CI)	Prevalence (95% CI)	Prevalence (95% CI)	Prevalence (95% CI)
Type of diabetes						
Type-1	16.1 (13.4, 19.2) ***	8.1 (6.1, 10.6) **	8.4 (6.3, 11.1) ***	0.9 (0.5, 1.7) *	2.7 (1.5, 5.0) ***	6.8 (5.2, 8.8) ***
Type-2	10.6 (10.1, 11.0)	5.3 (5.0, 5.7)	5.1 (4.8, 5.4)	0.4 (0.3, 0.5)	0.8 (0.7, 0.9)	4.0 (3.7, 4.3)
Duration of diabetes ^a^						
<5 years	6.1 (5.4, 6.8) ***	2.6 (2.2, 3.2) ***	3.4 (2.9, 4.0) ***	0.2 (0.1, 0.4) ***	0.3 (0.2, 0.6) ***	1.5 (1.2, 1.8) ***
5 to <10 years	8.6 (7.8, 9.6)	3.8 (3.2, 4.4)	4.0 (3.4, 4.8)	0.2 (0.1, 0.3)	0.4 (0.3, 0.7)	3.2 (2.6, 3.7)
10 to <15 years	10.5 (9.4, 11.7)	4.8 (4.1, 5.6)	4.6 (3.9, 5.4)	0.4 (0.3, 0.8)	0.6 (0.4, 1.0)	4.7 (4.0, 5.6)
15 years or more	17.0 (15.7, 18.3)	9.6 (8.6, 10.8)	7.9 (7.0, 8.9)	0.8 (0.6, 1.2)	2.1 (1.5, 2.7)	7.8 (7.0, 8.8)
BMI classification ^b^						
less than 25	20.2 (14.8, 27.0) ***	13.3 (9.0, 19.1) ***	8.2 (4.9, 13.4) ***	0.6 (0.1, 3.9) *	1.4 (0.4, 4.6)	7.0 (4.1, 11.9)
25 to less than 30	10.9 (9.9, 12.0)	6.5 (5.7, 7.3)	4.5 (3.9, 5.4)	0.7 (0.5, 1.2)	0.9 (0.6, 1.4)	3.9 (3.3, 4.6)
30 to less than 35	9.9 (9.2, 10.6)	4.4 (3.9, 4.9)	4.3 (3.8, 4.8)	0.4 (0.3, 0.6)	0.7 (0.6, 1.0)	4.3 (3.9, 4.9)
35 or more	11.1 (10.4, 11.8)	5.5 (5.0, 6.1)	6.0 (5.5, 6.5)	0.3 (0.2, 0.4)	0.9 (0.7, 1.2)	4.1 (3.7, 4.5)
High Blood Pressure						
Yes	10.9 (10.3, 11.5)	5.2 (4.8, 5.7)	5.1 (4.7, 5.6)	0.4 (0.3, 0.5)	0.9 (0.7, 1.1)	4.4 (4.1, 4.8) **
No	10.7 (10.0, 11.4)	5.7 (5.2, 6.2)	5.3 (4.8, 5.8)	0.5 (0.4, 0.7)	0.8 (0.6, 1.1)	3.7 (3.3, 4.1)
High Blood Cholesterol ^+^						
Yes	9.6 (8.9, 10.4) ***	4.6 (4.1, 5.1) ***	4.5 (4.0, 5.1) **	0.3 (0.2, 0.5)	0.7 (0.5, 1.0)	4.1 (3.6, 4.6)
No	11.3 (10.7, 11.8)	5.8 (5.4, 6.2)	5.5 (5.1, 5.9)	0.5 (0.4, 0.6)	0.9 (0.7, 1.1)	4.1 (3.8, 4.4)
Cardiovascular disease						
Yes	13.9 (13.3, 14.6) ***	7.0 (6.5, 7.5) ***	6.5 (6.0, 7.0) ***	0.5 (0.4, 0.6)	1.1 (0.9, 1.4) **	5.7 (5.3, 6.2) ***
No	7.4 (6.9, 8.0)	3.8 (3.4, 4.3)	3.8 (3.4, 4.3)	0.4 (0.2, 0.6)	0.6 (0.4, 0.8)	2.3 (2.0, 2.7)
Stroke						
Yes	16.6 (14.8, 18.5) ***	8.4 (7.1, 9.8) ***	7.9 (6.6, 9.3) ***	0.4 (0.2, 0.6)	1.3 (0.8, 2.1)	6.7 (5.6, 8.1) ***
No	10.2 (9.8, 10.7)	5.2 (4.8, 5.5)	5.0 (4.6, 5.3)	0.4 (0.3, 0.5)	0.8 (0.7, 1.0)	3.8 (3.6, 4.1)
Asthma						
Yes	12.7 (11.5, 14.1) ***	5.7 (4.9, 6.7)	7.4 (6.4, 8.5) ***	0.5 (0.3, 0.8)	0.7 (0.4, 1.2)	4.0 (3.3, 4.8)
No	10.5 (10.0, 11.0)	5.4 (5.0, 5.8)	4.9 (4.5, 5.2)	0.4 (0.3, 0.5)	0.9 (0.7, 1.1)	4.1 (3.8, 4.4)
Psychological distress ^^,a^						
Low	9.2 (8.7, 9.7) ***	4.6 (4.2, 5.0) **	4.4 (4.1, 4.9) ***	0.3 (0.2, 0.4) **	0.7 (0.5, 0.9)	3.2 (2.9, 3.6) ***
Moderate	11.5 (10.4, 12.7)	5.7 (4.9, 6.5)	5.9 (5.1, 6.8)	0.7 (0.5, 1.0)	1.0 (0.7, 1.4)	4.5 (3.8, 5.3)
High/very high	13.0 (11.5, 14.7)	6.0 (4.9, 7.2)	6.4 (5.3, 7.7)	0.7 (0.4, 1.1)	0.9 (0.5, 1.6)	5.9 (4.9, 7.2)

Notes: ***, **, and * denote *p*-values of 0.001, 0.01, and 0.05, respectively, for Pearson’s Chi-square test. Overlapping 95%CI indicated statistically non-significant. ^a^ Duration of diabetes has 25% missing values, whereas the missing value for psychological stress is 17.5%. ^b^ BMI has 1.5% missing values. ^+^ Participants were asked “In the last month have you been treated for high blood cholesterol?” ^^^ Psychological distress is categorised according to the Australian Bureau of Statistics classification. The Kessler-10 (K10) scale was used to categorise psychological distress into three groups: “low (10–15)”, “moderate (16–21)”, and “high/very high (22–50)”.

## Data Availability

For reasons of confidentiality, data from the 45 and the Up Study cohort and other related sources cannot be made public. The policies and procedures for data access can be found at www.saxinstitute.org.au, accessed on 25 September 2021.

## Appendix A

**Table A1:** ICD-10 AM codes for diabetes-related foot disease.

Complications	ICD-10 AM Codes
Insulin-dependent diabetes mellitus Non-insulin-dependent diabetes Mellitus Other specified diabetes mellitus Unspecified diabetes mellitus	E10 E11 E13 E14
Diabetes mellitus with foot ulcer	E1x.73
Ulcer of lower limb	L97
Chronic skin ulcer of lower limb	L98.4
Decubitus ulcer	L89
Atherosclerosis of arteries of extremities with ulceration/gangrene	I70.23/I70.24
Diabetes with peripheral angiopathy with gangrene	E1x.52
Subsidiary of gangrene	R02x
Cellulitis	L03.02, L03.11
Osteomyelitis	M86.x7, M86.x6
Procedure codes (lower limb amputations, including foot, toe, and ankle)	44370-00, 44373-00, 44367-01, 44367-02, 44338-00, 44358-00, 44361-00, 44361-01, 44364-00, 44364-01, 90557-00
Diabetic neuropathic arthropathy (Charcot)	E1x.61
Diabetic Mononeuropathy	G57, E1x.41
Diabetic Peripheral neuropathy	E1x.42
Diabetes with peripheral angiopathy without gangrene	E1x.51
Other Peripheral circulatory complication	I73.9

**Table A2:** ICD-9 CM codes for diabetes-related foot disease.

Complications	ICD 9 CM Codes
Diabetes	249, 250
Ulcer of lower limb	440.23, 707.1x
Gangrene of lower limb	440.24, 785.4
Arthropathy	250.8, 713, 713.5
Lower limb amputation	84.1, 84.10–17
Cellulitis	680, 680.7, 681, 681.1, 681.10, 681.11, 681.9, 682, 682.6, 682.7
Osteomyelitis	730, 730.0, 730.1, 730.2, 730.9
Peripheral Neuropathy	250.6, 357.2
Atherosclerosis	440, 440.20–24, 440.29
Peripheral vascular disease	250.7, 443.8, 443.9, 997.2

**Table A3:** Prevalence of DFD, DFU, DFI, DG, DLEA, and other DFD.

	Diabetes-Related Foot Disease (DFD) (*n* = 3035)	Diabetic Foot Ulcer (DFU) (*n* = 1503)	Diabetic Foot Infection (DFI) (*n* = 1448)	Diabetic Gangrene (DG) (*n* = 123)	Diabetes-Related Lower Extremity Amputation (DLEA) (*n* = 222)	Other Diabetes-Related Foot Disease (Other DFD) (*n* = 1189)
General population with diabetes (*n* = 28,210)	10.8	5.4	5.2	0.4	0.9	4.1
Inpatient population with diabetes (*n* = 20,029)	14.5	7.5	6.8	0.6	1.2	5.9
General population (*n* = 267,086)	1.2	0.6	0.6	0.05	0.09	0.5

**Table A4:** Characteristics of survey participants with diabetes (*n* = 28,210) according to different diabetic foot complications.

	Diabetes-Related Foot Disease (DFD)		Diabetic Foot Ulcer (DFU)		Diabetic Foot Infection (DFI)		Diabetic Gangrene (DG)		Diabetes-Related Lower Extremity Amputation (DLEA)		Other Diabetes-Related Foot Disease (Other DFD)	
	No (*n* = 25,175)	Yes (*n* = 3035)	No (*n* = 26,707)	Yes (*n* = 1503)	No (*n* = 26,762)	Yes (*n* = 1448)	No (*n* = 28,087)	Yes (*n* = 123)	No (*n* = 27,998)	Yes (*n* = 222)	No (*n* = 27,021)	Yes (*n* = 1189)
Age ^a^												
45–54 years	3781 (15.0%)	201 (6.6%)	3892 (14.6%)	90 (6.0%)	3867 (14.5%)	115 (7.9%)	3973 (14.2%)	9 (7.3%)	3963 (14.2%)	19 (8.6%)	3910 (14.5%)	72 (6.1%)
55–64 years	7411 (29.4%)	581 (19.1%)	7735 (29.0%)	257 (17.1%)	7671 (28.7%)	321 (22.2%)	7964 (28.4%)	28 (22.8%)	7932 (28.3%)	60 (27.0%)	7759 (28.7%)	233 (19.6%)
65–74 years	7709 (30.6%)	862 (28.4%)	8200 (30.7%)	371 (24.7%)	8171 (30.5%)	400 (27.6%)	8538 (30.4%)	33 (26.8%)	8514 (30.4%)	57 (25.7%)	8197 (30.3%)	374 (31.5%)
75+ years	6271 (24.9%)	1391 (45.8%)	6877 (25.8%)	785 (52.2%)	7050 (26.4%)	612 (42.3%)	7609 (27.1%)	53 (43.1%)	7576 (27.1%)	86 (38.7%)	7152 (26.5%)	510 (42.9%)
Gender												
Male	13,980 (55.5%)	1918 (63.2%)	14,953 (56.0%)	945 (62.9%)	15,006 (56.1%)	892 (61.6%)	15,800 (56.3%)	98 (79.7%)	15,735 (56.2%)	163 (73.4%)	15,090 (55.9%)	808 (68.0%)
Female	11,195 (44.5%)	1117 (36.8%)	11,754 (44.0%)	558 (37.1%)	11,756 (43.9%)	556 (38.4%)	12,287 (43.8%)	25 (20.3%)	12,253 (43.8%)	59 (26.6%)	11,931 (44.2%)	381 (32.0%)
Current Marital status ^b^												
Single	1577 (6.3%)	229 (7.6%)	1672 (6.3%)	134 (9.0%)	1669 (6.3%)	137 (9.5%)	1793 (6.4%)	13 (10.7%)	1785 (6.4%)	21 (9.6%)	1732 (6.5%)	74 (6.3%)
Married/defacto	17,750 (71.0%)	1814 (60.3%)	18,715 (70.6%)	849 (56.9%)	18,738 (70.6%)	826 (57.4%)	19,489 (69.9%)	75 (62.0%)	19,435 (70.0%)	129 (58.6%)	18,811 (70.1%)	753 (64.0%)
Widowed	3069 (12.3%)	608 (20.2%)	3345 (12.6%)	332 (22.3%)	3373 (12.7%)	304 (21.1%)	3657 (13.1%)	20 (16.5%)	3641 (13.1%)	36 (16.4%)	3469 (12.9%)	208 (17.7%)
Divorced/separated	2595 (10.4%)	358 (11.9%)	2776 (10.5%)	177 (11.9%)	2781 (10.5%)	172 (12.0%)	2940 (10.6%)	13 (10.7%)	2919 (10.5%)	34 (15.5%)	2812 (10.5%)	141 (12.0%)
Remoteness ^a,+^												
Major Cities	11,478 (45.6%)	1435 (47.3%)	12,173 (45.6%)	740 (49.2%)	12,255 (45.8%)	658 (45.4%)	12,854 (45.8%)	59 (48.0%)	12,813 (45.8%)	100 (45.1%)	12,362 (45.8%)	551(46.3%)
Regional	13,138 (52.2%)	1521 (50.1%)	13,937 (52.2%)	722 (48.0%)	13,915 (52.0%)	744 (51.4%)	14,603 (52.0%)	56 (45.5%)	14,545 (52.0%)	114 (51.4%)	14,043 (52.0%)	616 (51.8%)
Remote/Very Remote	558 (2.2%)	79 (2.6%)	596 (2.2%)	41 (2.7%)	591 (2.2%)	46 (3.2%)	629 (2.2%)	8 (6.5%)	629 (2.3%)	8 (3.6%)	615 (2.2%)	22 (1.9%)
Born in Australia ^b^												
Yes	17,957 (72.4%)	2254 (75.6%)	19,083 (72.5%)	1128 (76.3%)	19,110 (72.5%)	1101 (77.2%)	20,120 (72.7%)	91 (75.8%)	20,030 (72.7%)	181 (82.7%)	19,341 (72.6%)	870 (74.6%)
No	6853 (27.6%)	727 (24.4%)	7229 (27.5%)	351 (23.7%)	7255 (27.5%)	325 (22.8%)	7551 (27.3%)	29 (24.2%)	7542 (27.4%)	38 (17.4%)	7283 (27.4%)	297 (25.5%)
Language spoken other than English												
Yes	3394 (13.5%)	319 (10.5%)	3561 (13.3%)	152 (10.1%)	3561 (13.3%)	152 (10.5%)	3698 (13.2%)	15 (12.2%)	3692 (13.2%)	21 (9.5%)	3588 (13.3%)	125 (10.5%)
No	21,781 (86.5%)	2716 (89.5%)	23,146 (86.7%)	1351 (89.9%)	23,201 (86.7%)	1296 (89.5%)	24,389 (86.8%)	108 (87.8%)	24,296 (86.8%)	201 (90.5%)	23,433 (86.7%)	1064 (89.5%)
Education ^b^												
Less than high school	10,570 (43.0%)	1456 (49.8%)	11,290 (43.4%)	736 (51.1%)	11,299 (43.3%)	727 (52.1%)	11,966 (43.7%)	60 (51.7%)	11,913 (43.7%)	113 (52.3%)	11,487 (43.6%)	539 (46.9%)
High school certificate/Trade	5677 (23.1%)	696 (23.8%)	6013 (23.1%)	360 (25.0%)	6077 (23.3%)	296 (21.2%)	6345 (23.2%)	28 (24.1%)	6317 (23.2%)	56 (25.9%)	6057 (23.0%)	316 (27.5%)
Certificate/diploma	4351 (17.7%)	452 (15.5%)	4601 (17.7%)	202 (14.0%)	4581 (17.6%)	222 (15.9%)	4785 (17.5%)	18 (15.5%)	4775 (17.5%)	28 (13.0%)	4624 (17.6%)	179 (15.6%)
University degree or higher	3963 (16.1%)	322 (11.0%)	4142 (15.9%)	143 (9.9%)	4135 (15.9%)	150 (10.8%)	4275 (15.6%)	10 (8.6%)	4266 (15.6%)	19 (8.8%)	4170 (15.8%)	115 (10.0%)
SEIFA (IRSD) ^^,a^												
quantile 1 (least disadvantaged)	5101 (20.3%)	621 (20.5%)	5415 (20.3%)	307 (20.4%)	5396 (20.2%)	326 (22.5%)	5695 (20.3%)	27 (22.0%)	5670 (20.3%)	52 (23.4%)	5505 (20.4%)	217 (18.3%)
quantile 2	5229 (20.8%)	643 (21.2%)	5551 (20.8%)	321 (21.4%)	5572 (20.8%)	300 (20.7%)	5846 (20.8%)	26 (21.1%)	5833 (20.8%)	39 (17.6%)	5614 (20.8%)	258 (21.7%)
quantile 3	5330 (21.2%)	645 (21.3%)	5651 (21.2%)	324 (21.6%)	5667 (21.2%)	308 (21.3%)	5953 (21.2%)	22 (17.9%)	5918 (21.2%)	57 (25.7%)	5692 (21.1%)	283 (23.8%)
quantile 4	4524 (18.0%)	515 (17.0%)	4796 (18.0%)	243 (16.2%)	4812 (18.0%)	227 (15.7%)	5019 (17.9%)	20 (16.3%)	5000 (17.9%)	39 (17.6%)	4837 (17.9%)	202 (17.0%)
quantile 5 (most disadvantaged)	4988 (19.8%)	611 (20.1%)	5291 (19.8%)	308 (20.5%)	5312 (19.9%)	287 (19.8%)	5571 (19.8%)	28 (22.8%)	5564 (19.9%)	35 (15.8%)	5370 (19.9%)	229 (19.3%)
Annual Household income (in AUD) ^c^												
<20 K	7756 (33.0%)	1239 (45.9%)	8374 (33.6%)	621 (46.5%)	8389 (33.6%)	606 (47.3%)	8941 (34.2%)	54 (46.6%)	8900 (34.2%)	95 (49.2%)	8523 (33.8%)	472 (45.5%)
20 K–<50 K	6264 (26.6%)	639 (23.7%)	6589 (26.5%)	314 (23.5%)	6616 (26.5%)	287 (22.4%)	6870 (26.3%)	33 (28.5%)	6861 (26.4%)	42 (21.8%)	6640 (26.4%)	263 (25.3%)
>50 K	5067 (21.5%)	304 (11.3%)	5235 (21.0%)	136 (10.2%)	5216 (20.9%)	155 (12.1%)	5364 (20.5%)	7 (6.0%)	5355 (20.6%)	16 (8.3%)	5272 (20.9%)	99 (9.5%)
did not disclose	4445 (18.9%)	515 (19.1%)	4696 (18.9%)	264 (19.8%)	4727 (19.0%)	233 (18.2%)	4938 (18.9%)	22 (19.0%)	4920 (18.9%)	40 (20.7%)	4756 (18.9%)	204 (19.7%)
Work Status^^^,b^												
Paid	7585 (30.6%)	380 (12.8%)	7825 (29.7%)	140 (9.6%)	7762 (29.4%)	203 (14.3%)	7950 (28.7%)	15 (12.5%)	7937 (28.8%)	28 (13.0%)	7823 (29.4%)	142 (12.1%)
Retired	13,529 (54.5%)	2075 (70.0%)	14,528 (55.2%)	1076 (73.5%)	14,656 (55.6%)	948 (67.0%)	15,527 (56.1%)	77 (64.2%)	15,464 (56.1%)	140 (65.1%)	14,775 (55.5%)	829 (70.8%)
Other	3707 (14.9%)	511 (17.2%)	3969 (15.1%)	249 (17.0%)	3953 (15.0%)	265 (18.7%)	4190 (15.1%)	28 (23.3%)	4171 (15.1%)	47 (21.9%)	4018 (15.1%)	200 (17.1%)
Private Health insurance												
Yes	13,179 (52.4%)	1262 (41.6%)	13,841 (51.8%)	600 (39.9%)	13,878 (51.9%)	563 (38.9%)	14,397 (51.3%)	44 (35.8%)	14,373 (51.4%)	68 (30.6%)	13,959 (51.7%)	482 (40.5%)
No (without DVA and Concession card) ^^^^^	3866 (15.4%)	453 (14.9%)	4102 (15.4%)	217 (14.4%)	4076 (15.2%)	243 (16.8%)	4296 (15.3%)	23 (18.7%)	4286 (15.3%)	33 (14.9%)	4148 (15.4%)	171 (14.4%)
No (with DVA/concession card)	8130 (32.3%)	1320 (43.5%)	8764 (32.8%)	686 (45.6%)	8808 (32.9%)	642 (44.3%)	9394 (33.5%)	56 (45.5%)	9329 (33.3%)	121 (54.5%)	8914 (33.0%)	536 (45.1%)
Ever being a regular smoker ^a^												
Yes	12,188 (48.4%)	1714 (56.6%)	13,063 (48.9%)	839 (55.9%)	13,111 (49.0%)	791 (54.8%)	13,830 (49.3%)	72 (58.5%)	13,778 (49.3%)	124 (56.1%)	13,165 (48.8%)	737 (62.0%)
No	12,976 (51.6%)	1317 (43.5%)	13,632 (51.1%)	661 (44.1%)	13,640 (51.0%)	653 (45.2%)	14,242 (50.7%)	51 (41.5%)	14,196 (50.8%)	97 (43.9%)	13,841 (51.3%)	452 (38.0%)
Alcohol consumption ^b^												
< = 14 drinks per week	21,507 (88.6%)	2590 (89.0%)	22,813 (88.6%)	1284 (89.0%)	22,867 (88.7%)	1230 (88.7%)	23,996 (88.7%)	101 (84.9%)	23,912 (88.7%)	185 (86.1%)	23,101 (88.7%)	996 (87.7%)
>14 drinks per week	2762 (11.4%)	320 (11.0%)	2923 (11.4%)	159 (11.0%)	2926 (11.3%)	156 (11.3%)	3064 (11.3%)	18 (15.1%)	3052 (11.3%)	30 (14.0%)	2942 (11.3%)	140 (12.3%)
Total moderate-to-vigorous physical activity per week (minutes)												
<150 min	8684 (34.5%)	1581 (52.1%)	9418 (35.3%)	847 (56.4%)	9524 (35.6%)	741 (51.2%)	10,198 (36.3%)	67 (54.5%)	10,146 (36.3%)	119 (53.6%)	9631 (35.6%)	634 (53.3%)
150–300 min	3866 (15.4%)	393 (13.0%)	4070 (15.2%)	189 (12.6%)	4074 (15.2%)	185 (12.8%)	4238 (15.1%)	21 (17.1%)	4232 (15.1%)	27 (12.2%)	4095 (15.2%)	164 (13.8%)
>300 min	12,625 (50.2%)	1061 (35.0%)	13,219 (49.5%)	467 (31.1%)	13,164 (49.2%)	522 (36.1%)	13,651 (48.6%)	35 (28.5%)	13,610 (48.6%)	76 (34.2%)	13,295 (49.2%)	391 (32.9%)
Vegetables intake ^b^												
<5 serves per day	16,079 (65.3%)	1953 (67.2%)	17,064 (65.4%)	968 (67.6%)	17,121 (65.5%)	911 (66.1%)	17,944 (65.5%)	88 (72.7%)	17,896 (65.5%)	136 (65.4%)	17,265 (65.4%)	767 (67.1%)
5 or more serves per day	8541 (34.7%)	954 (32.8%)	9030 (34.6%)	465 (32.5%)	9027 (34.5%)	468 (33.9%)	9462 (34.5%)	33 (27.3%)	9423 (34.5%)	72 (34.6%)	9119 (34.6%)	376 (32.9%)
Fruit intake ^b^												
<2 serves per day	9842 (39.6%)	1248 (41.7%)	10,467 (39.7%)	623 (42.0%)	10,504 (39.8%)	586 (41.3%)	11,033 (39.8%)	57 (47.5%)	10,991 (39.8%)	99 (45.8%)	10,606 (39.8%)	484 (41.3%)
2 or more serves per day	14,993 (60.4%)	1742 (58.3%)	15,876 (60.3%)	859 (58.0%)	15,902 (60.2%)	833 (58.7%)	16,672 (60.2%)	63 (52.5%)	16,618 (60.2%)	117 (54.2%)	16,048 (60.2%)	687 (58.7%)
Types of diabetes												
Type 1	826 (3.3%)	157 (5.2%)	906 (3.4%)	77 (5.1%)	910 (3.4%)	73 (5.0%)	971 (3.5%)	12 (9.8%)	964 (3.4%)	19 (8.6%)	909 (3.4%)	74 (6.2%)
Type 2	24,349 (96.7%)	2878 (94.8%)	25,801 (96.6%)	1426 (94.9%)	25,852 (96.6%)	1375 (95.0%)	27,116 (96.5%)	111 (90.2%)	27,024 (96.6%)	203 (91.4%)	26,112 (96.6%)	1115 (93.8%)
Duration of diabetes ^c^												
<5 years	6626 (34.9%)	432 (20.3%)	6874 (34.2%)	184 (17.9%)	6823 (33.9%)	235 (23.4%)	7043 (33.5%)	15 (17.1%)	7040 (33.5%)	18 (11.5%)	6934 (34.2%)	124 (14.1%)
5 to <10 years	5020 (26.4%)	464 (21.8%)	5274 (26.2%)	210 (20.5%)	5276 (26.2%)	208 (20.7%)	5470 (26.0%)	14 (15.9%)	5456 (26.0%)	28 (17.8%)	5309 (26.2%)	175 (19.9%)
10 to <15 years	3381 (17.8%)	430 (20.2%)	3610 (17.9%)	201 (19.6%)	3611 (17.9%)	200 (19.9%)	3794 (18.0%)	17 (19.3%)	3782 (18.0%)	29 (18.5%)	3624 (17.9%)	187 (21.2%)
15 years or more	3988 (21.0%)	803 (37.7%)	4360 (21.7%)	431 (42.0%)	4429 (22.0%)	362 (36.0%)	4749 (22.6%)	42 (47.7%)	4709 (22.4%)	82 (52.2%)	4396 (21.7%)	395 (44.8%)
BMI classification ^b^												
<25	175 (0.7%)	41 (1.4%)	188 (0.7%)	28 (1.9%)	200 (0.8%)	16 (1.1%)	215 (0.8%)	-	213 (0.8%)	-	202 (0.8%)	14 (1.2%)
25 to <30	4757 (19.2%)	582 (19.6%)	5013 (19.1%)	326 (22.2%)	5110 (19.4%)	229 (16.2%)	5303 (19.2%)	36 (29.5%)	5300 (19.2%)	39 (18.0%)	5124 (19.3%)	215 (18.2%)
30 to <35	8612 (34.7%)	946 (31.9%)	9129 (34.7%)	429 (29.3%)	9153 (34.7%)	405 (28.7%)	9524 (34.4%)	34 (27.9%)	9486 (34.4%)	72 (33.2%)	9148 (34.4%)	410 (34.7%)
35 or more	11,269 (45.4%)	1401 (47.2%)	11,987 (45.6%)	683 (46.6%)	11,908 (45.2%)	762 (54.0%)	12,619 (45.6%)	51 (41.8%)	12,567 (45.6%)	103 (47.5%)	12,128 (45.6%)	542 (45.9%)
High Blood Pressure												
Yes	13,699 (54.4%)	1708 (56.3%)	14,596 (54.7%)	811 (54.0%)	14,599 (54.6%)	808 (55.8%)	15,345 (54.6%)	62 (50.4%)	15,272 (54.6%)	135 (60.8%)	14,700 (54.4%)	707 (59.5%)
No	11,476 (45.6%)	1327 (43.7%)	12,111 (45.4%)	692 (46.0%)	12,163 (45.5%)	640 (44.2%)	12,742 (45.4%)	61 (49.6%)	12,716 (45.4%)	87 (39.2%)	12,321 (45.6%)	482 (40.5%)
High Blood Cholesterol ^⸶^												
Yes	7623 (30.3%)	823 (27.1%)	8063 (30.2%)	383 (25.5%)	8065 (30.1%)	381 (26.3%)	8418 (30.0%)	28 (22.8%)	8390 (30.0%)	56 (25.2%)	8087 (29.9%)	359 (30.2%)
No	17,552 (69.7%)	2212 (72.9%)	18,644 (69.8%)	1120 (74.5%)	18,697 (69.9%)	1067 (73.7%)	19,669 (70.0%)	95 (77.2%)	19,598 (70.0%)	166 (74.8%)	18,934 (70.1%)	830 (69.8%)
Cardiovascular disease												
Yes	12,939 (51.4%)	2055 (67.7%)	13,990 (52.4%)	1004 (66.8%)	14,037 (52.5%)	957 (66.1%)	14,914 (53.1%)	80 (65.0%)	14,840 (53.0%)	154 (69.4%)	14,121 (52.3%)	873 (73.4%)
No	12,236 (48.6%)	980 (32.3%)	12,717 (47.6%)	499 (33.2%)	12,725 (47.6%)	491 (33.9%)	13,173 (46.9%)	43 (35.0%)	13,148 (47.0%)	68 (30.6%)	12,900 (47.7%)	316 (26.6%)
Stroke												
Yes	1928 (7.7%)	385 (12.7%)	2115 (7.9%)	198 (13.2%)	2136 (8.0%)	177 (12.2%)	2298 (8.2%)	15 (12.2%)	2283 (8.2%)	30 (13.5%)	2159 (8.0%)	154 (13.0%)
No	23,247 (92.3%)	2650 (87.3%)	24,592 (92.1%)	1305 (86.8%)	24,626 (92.0%)	1271 (87.8%)	25,789 (91.8%)	108 (87.8%)	25,705 (91.8%)	192 (86.5%)	24,862 (92.0%)	1035 (87.1%)
Asthma												
Yes	3332 (13.2%)	467 (15.4%)	3590 (13.4%)	209 (13.9%)	3529 (13.2%)	270 (18.7%)	3781 (13.5%)	18 (14.6%)	3774 (13.5%)	25 (11.3%)	3643 (13.5%)	156 (13.1%)
No	21,843 (86.8%)	2568 (84.6%)	23,117 (86.6%)	1294 (86.1%)	23,233 (86.8%)	1178 (81.4%)	24,306 (86.5%)	105 (85.4%)	24,214 (86.5%)	197 (88.7%)	23,378 (86.5%)	1033 (86.9%)
Psychological distress ^§,c^												
Low	14,799 (70.8%)	1504 (63.2%)	15,570 (70.4%)	733 (63.5%)	15,594 (70.4%)	709 (61.8%)	16,255 (70.1%)	48 (48.0%)	16,199 (70.1%)	104 (59.8%)	15,741 (70.4%)	562 (59.8%)
Moderate	3735 (17.9%)	505 (21.2%)	3986 (18.0%)	254 (22.0%)	3980 (18.0%)	260 (22.7%)	4205 (18.1%)	35 (35.0%)	4193 (18.1%)	47 (27.0%)	4035 (18.1%)	205 (21.8%)
* High/very high*	2371 (11.3%)	372 (15.6%)	2575 (11.6%)	168 (14.6%)	2564 (11.6%)	179 (15.6%)	2726 (11.8%)	17 (17.0%)	2720 (11.8%)	23 (13.2%)	2570 (11.5%)	173 (18.4%)

Notes: - small cell. ^a^ Less than 1% missing values for *age, marital status, remoteness, SEIFA, and ever being a smoker*. ^b^ Less than 4% missing values for *born in Australia, work status, fruit intake, vegetable intake, alcohol consumption, and BMI*. ^c^ *Annual household income* has 25% missing values, including “did not disclose”. *Duration of diabetes* has 25% missing values, whereas the missing value for *psychological stress* is 17.5%. ^+^ Remoteness of residence is derived from the Accessibility-Remoteness Index of Australia scores from 2006 (Australian Government Department of Health). These scores were calculated based upon distance by road to the nearest population centre where services can be obtained. The Australian Bureau of Statistics uses this score to classify different regions into major cities, inner regional, outer regional, remote, and very remote. ^^^ Socioeconomic status was assessed by index of relative socioeconomic disadvantage (IRSD), which is one of indices of the Socioeconomic Indexes for Areas (SEIFA) (Australian Bureau of Statistics). ^^^^ “Other” category in work status included individuals who were sick, disabled, students, or unemployed. ^^^^^ DVA denotes Department of Veterans’ Affairs. DVA or concession card holders may receive higher discounted health services and medication. ^⸶^ Participants were asked, “In the last month have you been treated for high blood cholesterol?” ^§^ Psychological distress is categorised according to the Australian Bureau of Statistics classification. The Kessler-10 (K10) scale was used to categorise psychological distress into three groups: “low (10–15)”, “moderate (16–21)”, and “high/very high (22–50)”.

**Table A5:** Prevalence (unweighted) of DFD, DFU, DFI, DG, DLEA, and other DFD among people with diabetes in terms of demographic, socioeconomic, and lifestyle risk factors.

	Diabetes-Related Foot Disease (DFD) (*n* = 3035)	Diabetic Foot Ulcer (DFU) (*n* = 1503)	Diabetic Foot Infection (DFI) (*n* = 1448)	Diabetic Gangrene (DG) (*n* = 123)	Diabetes-Related Lower Extremity Amputation (DLEA) (*n* = 222)	Other Diabetes-Related Foot Disease (Other DFD) (*n* = 1189)
Overall	10.8	5.3	5.1	0.4	0.8	4.2
**Demographic factors**						
Age ^a^						
45–54 years	5.0	2.3	2.9	0.2	0.5	1.8
55–64 years	7.3	3.2	4.0	0.4	0.8	2.9
65–74 years	10.1	4.3	4.7	0.4	0.7	4.4
75+ years	18.2	10.2	8.0	0.7	1.1	6.7
Gender						
Male	12.1	5.9	5.6	0.6	1.0	5.1
Female	9.1	4.5	4.5	0.2	0.5	3.1
Current marital status ^a^						
Single	12.7	7.4	7.6	0.7	1.2	4.1
Married/defacto	9.3	4.3	4.2	0.4	0.7	3.8
Widowed	16.5	9.0	8.3	0.5	1.0	5.7
Divorced/separated	12.1	6.0	5.8	0.4	1.2	4.8
Remoteness ^+,a^						
Major Cities	11.1	5.7	5.1	0.5	0.8	4.3
Regional	10.4	4.9	5.1	0.4	0.8	4.2
Remote/Very remote	12.4	6.4	7.2	1.3	1.3	3.5
Born in Australia ^b^						
Yes	11.2	5.6	5.4	0.5	0.9	4.3
No	9.6	4.6	4.3	0.4	0.5	3.9
Language spoken other than English						
Yes	8.6	4.1	4.1	0.4	0.6	3.4
No	11.1	5.5	5.3	0.4	0.8	4.3
**Socioeconomic factors**						
Education ^b^						
Less than high school	12.1	6.1	6.0	0.5	0.9	4.5
High school certificate/trade	10.9	5.6	4.6	0.4	0.9	5.0
Certificate/diploma	9.4	4.2	4.6	0.4	0.6	3.7
University degree or higher	7.5	3.3	3.5	0.2	0.4	2.7
SEIFA (IRSD) ^^,a^						
quantile 1 (least disadvantaged)	10.9	5.4	5.7	0.5	0.9	3.8
quantile 2	11.0	5.5	5.1	0.4	0.7	4.4
quantile 3	10.8	5.4	5.2	0.4	1.0	4.7
quantile 4	10.2	4.8	4.5	0.4	0.8	4.0
quantile 5 (most disadvantaged)	10.9	5.5	5.1	0.5	0.6	4.1
Annual household income (in AUD) ^c^						
<20 K	13.8	6.9	6.7	0.6	1.1	5.2
20 K–<$50 K	9.3	4.5	4.2	0.5	0.6	3.8
>50 K	5.7	2.5	2.9	0.1	0.3	1.8
did not disclose	10.4	5.3	4.7	0.4	0.8	4.1
Work Status ^^^,b^						
Paid	4.8	1.8	2.5	0.2	0.4	1.8
Retired	13.3	6.9	6.1	0.5	0.9	5.3
Other	12.1	5.9	6.3	0.7	1.1	4.6
Private Health insurance						
Yes	7.8	3.8	3.6	0.3	0.5	2.9
No (without DVA/concession card) ^^^^^	11.5	5.7	6.0	0.5	0.9	4.6
No (with DVA/concession card)	12.9	6.5	6.1	0.5	1.0	5.1
**Lifestyle risk factors**						
Ever being a regular smoker ^a^						
Yes	12.3	6.0	5.7	0.5	0.9	5.3
No	9.2	4.6	4.6	0.4	0.7	3.2
Alcohol consumption ^b^						
≤14 drinks per week	10.7	5.3	5.1	0.4	0.8	4.1
>14 drinks per week	10.4	5.2	5.1	0.6	1.0	4.5
Total moderate-to-vigorous physical activity per week (minutes)						
<150 min	15.4	8.3	7.2	0.7	1.2	6.2
150–300 min	9.2	4.4	4.3	0.5	0.6	3.9
>300 min	7.8	3.4	3.8	0.3	0.6	2.9
Vegetables intake ^b^						
<5 serves per day	10.8	5.4	5.1	0.5	0.8	4.3
5 or more serves per day	1	4.9	4.9	0.3	0.8	4.0
Fruit intake ^b^						
<2 serves per day	11.3	5.6	5.3	0.5	0.9	4.4
2 or more serves per day	10.4	5.1	5.0	0.4	0.7	4.1
**Health status factors**						
Type of diabetes						
Type-1	16.0	7.8	7.4	1.2	1.9	7.5
Type-2	10.6	5.2	5.1	0.4	0.7	4.1
Duration of diabetes ^c^						
<5 years	6.1	2.6	3.3	0.2	0.3	1.8
5 to <10 years	8.5	3.8	3.8	0.3	0.5	3.2
10 to <15 years	11.3	5.3	5.2	0.4	0.8	4.9
15 years or more	16.8	9.0	7.6	0.9	1.7	8.2
BMI classification ^b^						
less than 25	11.2	6.4	4.4	0.7	0.8	4.1
25 to less than 30	9.9	4.5	4.2	0.4	0.8	4.3
30 to less than 35	9.1	4.5	4.5	0.4	0.6	3.9
35 or more	13.0	6.3	7.6	0.4	1.0	4.6
High Blood Pressure						
Yes	11.1	5.3	5.2	0.4	0.9	4.6
No	10.4	5.4	5.0	0.5	0.7	3.8
High Blood Cholesterol ^⸶^						
Yes	9.7	4.5	4.5	0.3	0.7	4.3
No	11.2	5.7	5.4	0.5	0.8	4.2
Cardiovascular disease						
Yes	13.7	6.7	6.4	0.5	1.0	5.8
No	7.4	3.8	3.7	0.3	0.5	2.4
Stroke						
Yes	16.6	8.6	7.7	0.6	1.3	6.7
No	10.2	5.0	4.9	0.4	0.7	4.0
Asthma						
Yes	12.3	5.5	7.1	0.5	0.7	4.1
No	10.5	5.3	4.8	0.4	0.8	4.2
Psychological distress ^^,a, §^						
Low	9.2	4.5	4.3	0.3	0.6	3.4
Moderate	11.9	6.0	6.1	0.8	1.1	4.8
High/very high	13.6	6.1	6.5	0.6	0.8	6.2

Notes: ^a^ Less than 1% missing values for *age, marital status, remoteness, SEIFA, and ever being a smoker*. ^b^ Less than 4% missing values for *born in Australia, work status, fruit intake, vegetable intake, alcohol consumption, and BMI*. ^c^ *Annual household income* has 25% missing values, including “did not disclose”. *Duration of diabetes* has 25% missing values, whereas the missing value for *psychological stress* is 17.5%. ^+^ Remoteness of residence is derived from the Accessibility-Remoteness Index of Australia scores from 2006 (Australian Government Department of Health). These scores were calculated based upon distance by road to the nearest population centre where services can be obtained. The Australian Bureau of Statistics uses this score to classify different regions into major cities, inner regional, outer regional, remote, and very remote. ^^^ Socioeconomic status was assessed by index of relative socioeconomic disadvantage (IRSD), which is one of indices of the Socioeconomic Indexes for Areas (SEIFA) (Australian Bureau of Statistics). ^^^^ “Other” category in work status included individuals who were sick, disabled, students, or unemployed. ^^^^^ DVA denotes Department of Veterans’ Affairs. DVA or concession card holders may receive higher discounted health services and medication. ^⸶^ Participants were asked, “In the last month have you been treated for high blood cholesterol?” ^§^ Psychological distress is categorised according to the Australian Bureau of Statistics classification. The Kessler-10 (K10) scale was used to categorise psychological distress into three groups: “low (10–15)”, “moderate (16–21)”, and “high/very high (22–50)”.

**Table A6:** Age-standardised ^^^ prevalence of diabetic foot complications among people with diabetes in different LHDs of NSW state among individuals aged 45 years and over during 2006–2012.

Local Health District (LHD)	Diabetes-Related Foot Disease (DFD)	Diabetic Foot Ulcer (DFU)	Diabetic Foot Infection (DFI)	Diabetic Gangrene (DG)	Diabetes-Related Lower Extremity Amputation (DLEA)	Other Diabetes-Related Foot Disease (Other DFD)
Central Coast	10.9	5.6	0.6	0.3	0.6	5.0
Hunter New England	10.7	5.1	4.4	0.5	0.8	4.4
Illawarra Shoalhaven	7.9	3.3	4.1	0.1	0.5	3.1
Mid North Coast	8.2	4.3	4.1	0.5	0.6	3.0
Murrumbidgee	10.5	4.7	5.5	0.5	1.0	4.7
Nepean Blue Mountains	10.4	5.0	5.4	0.6	0.7	3.2
Northern NSW	8.5	4.5	4.4	0.4	1.0	3.1
Northern Sydney	7.0	3.8	4.0	0.3	0.5	2.4
South Eastern Sydney	9.1	4.5	4.3	0.3	0.8	3.1
South Western Sydney	8.8	4.2	4.2	0.2	0.5	2.8
Southern NSW	8.3	3.2	4.9	0.6	0.2	2.9
Sydney	9.6	5.0	5.4	0.6	0.9	3.8
Western NSW	8.6	4.5	5.2	0.8	1.0	2.9
Western Sydney	10.2	5.4	6.0	0.2	1.4	3.5
Far West	13.9	7.8	6.0	0.4	0.4	3.1

Note: ^^^ Age standardised to the 2001 Australian standard population [19].
